# Microbiological screenings for infection control in unaccompanied minor refugees: the German Armed Forces Medical Service’s experience

**DOI:** 10.1186/s40779-017-0123-8

**Published:** 2017-04-21

**Authors:** Winfried Maaßen, Dorothea Wiemer, Claudia Frey, Christina Kreuzberg, Egbert Tannich, Rebecca Hinz, Andreas Wille, Andreas Fritsch, Ralf Matthias Hagen, Hagen Frickmann

**Affiliations:** 1 Department of Tropical Medicine at the Bernhard Nocht Institute, German Armed Forces Hospital of Hamburg, Bernhard Nocht Street 74, D-20359 Hamburg, Germany; 2 German Society for International Cooperation (GIZ), Bonn, Germany; 3 Department of Molecular Parasitology, Bernhard Nocht Institute for Tropical Medicine Hamburg, Hamburg, Germany; 4 Institute of Medical Microbiology, Virology and Hygiene, University Medical Center Hamburg-Eppendorf, Hamburg, Germany; 5 Institute for Hygiene and Environment, Hamburg, Germany; 6 Department of Laboratory Medicine, German Armed Forces Hospital of Hamburg, Hamburg, Germany; 7 NATO Center of Excellence for Military Medicine (MilMedCOE), Deployment Health Surveillance Capability (DHSC), Munich, Germany; 8 Institute for Microbiology, Virology and Hygiene, University Medicine Rostock, Rostock, Germany

**Keywords:** Refugee, Migration, Asylum seeker, Infection control, Screening, Gastrointestinal pathogens

## Abstract

**Background:**

The German Military Medical Service contributed to the medical screening of unaccompanied minor refugees (UMRs) coming to Germany in 2014 and 2015. In this study, a broad range of diagnostic procedures was applied to identify microorganisms with clinical or public health significance. Previously, those tests had only been used to screen soldiers returning from tropical deployments. This instance is the first time the approach has been studied in a humanitarian context.

**Methods:**

The offered screenings included blood cell counts, hepatitis B serology and microscopy of the stool to look for protozoa and worm eggs as well as PCR from stool samples targeting pathogenic bacteria, protozoa and helminths. If individuals refused certain assessments, their decision to do so was accepted. A total of 219 apparently healthy male UMRs coming from Afghanistan, Egypt, Somalia, Eritrea, Syria, Ghana, Guinea, Iran, Algeria, Iraq, Benin, Gambia, Libya, Morocco, Pakistan, and Palestine were assessed. All UMRs who were examined at the study department were included in the assessment.

**Results:**

We detected decreasing frequencies of pathogens that included diarrhoea-associated bacteria [C*ampylobacter (C.) jejuni*, enteropathogenic *Escherichia (E.) coli* (EPEC), enterotoxic *E. coli* (ETEC), enteroaggregative *E. coli* (EAEC), enteroinvasive *E. coli* (EIEC)/*Shigella* spp.), *Giardia (G.) duodenalis*, helminths (comprising *Schistosoma* spp., *Hymenolepis (H.) nana*, *Strongyloides (S.) stercoralis*] as well as hepatitis B virus. Pathogenic microorganisms dominated the samples by far. While *G. duodenalis* was detected in 11.4% of the assessed UMRs, the incidence of newly identified cases in the German population was 4.5 cases per 100,000 inhabitants.

**Conclusions:**

We conclude that the applied in-house PCR screening systems, which have proven to be useful for screening military returnees from tropical deployments, can also be used for health assessment of immigrants from the respective sites. Apparently healthy UMRs may be enterically colonized with a broad variety of pathogenic and apathogenic microorganisms. Increased colonization rates, as shown for *G. duodenalis,* can pose a hygiene problem in centralized homes for asylum seekers.

## Background

As a consequence of the recent political changes and military crises in the Middle East and Northern Africa, there has been an influx of millions of refugees into Europe, which has led to a considerable challenge for the civilian public health system. Therefore, since 2013, support from the military medical services in Germany has become necessary. This resulted in an agreement between the public health service of the Hanseatic city of Hamburg and the German Armed Forces Hospital of Hamburg (BwKH) for military physicians at the Department of Tropical Medicine at the Bernhard Nocht Institute for Tropical Medicine (DTM-BNITM) to perform health checks of unaccompanied minor refugees (UMRs).

According to § 36 of the German Act for the prevention and control of infectious diseases in humans (Infektionsschutzgesetz, IfSG), refugees shall undergo a health check when they arrive at a reception centre. These health checks are intended to exclude infectious diseases, which are likely to spread easily in vulnerable populations, such as those in reception centres, therefore posing a considerable hygiene problem. At the same time, primary and booster immunizations are administered according to the recommendations of the German Standing Committee on Vaccination (STIKO), which includes Tdap-IPV (tetanus, diphtheria, poliomyelitis, pertussis) and MMR (measles, mumps, rubella) vaccines.

At the Department of Tropical Medicine at the Bernhard Nocht Institute (DTM-BNITM), several in-house PCR systems have been established in order to screen soldiers returning from sub-tropical and tropical deployment settings for gastrointestinal pathogens [1–5]. These screening tests were applied, in addition to standard microscopic and serological examinations to gain deeper insight into infections, colonization and infestations with potentially hygiene-relevant pathogens. The results of this approach in UMRs are reported in the following sections.

## Methods

### Refugees

UMRs, defined as refugees of less than 18 years of age, were sent to the DTM-BNITM, BwKH, by the public health authority of Hamburg between September 2014 and December 2015. A chest X-ray was performed by the public health authorities to exclude active tuberculosis prior to the presentation of the UMRs to the DTM-BNITM. Military physicians documented the medical history of the UMRs with the help of an interpreter, examined them, took the necessary blood samples for laboratory testing and finally administered vaccines for tetanus, diphtheria, poliomyelitis, pertussis, measles, German measles (rubella), and mumps. UMR attendants were asked to send stool specimens of the UMRs after the consultation. Material for sample collection was provided with an explanation on how to use it.

There was no specific sampling method for the inclusion of UMRs in the study. All UMRs that were examined at the DTM-BNITM, BwKH, were included in the descriptive assessment without exemptions. There was no exclusion criterion for this descriptive assessment.

### Infectious disease screenings

Blood cell counts were performed at the BNITM laboratories in Hamburg, Germany, or the BwKH in Hamburg, Germany, using a Cell Dyn 3200 device (Abbott, Chicago, Illinois, USA). Serology for hepatitis B [anti-HBc-(hepatitis B core-) antibodies, HBs-(hepatitis B surface-) antigen] was performed at the Institute for Hygiene and Environment in the City of Hamburg, Germany, using an Architect i1000 immunology analyser (Abbott). Blood analyses were based on standardized procedures in quality-controlled diagnostic laboratories.

Stool samples were either formalin-fixed [6] to look for protozoa and helminth eggs using microscopy at the BNITM in Hamburg, Germany, or the samples were used for DNA extraction without any additives. The PCR protocols used comprised both in-house and commercial approaches. The in-house approaches covered enteroinvasive bacteria (*Salmonella* spp., EIEC (enteroinvasive *Escherichia* (*E.*) *coli*)/*Shigella* spp., *Campylobacter (C.) jejuni*, and *Yersinia* spp.) in one single-tube multiplex real-time assay [1, 2] and enteropathogenic protozoa (*Entamoeba (E.) histolytica*, *Giardia (G.) duodenalis*, *Cryptosporidium (C.) parvum* and *Cyclospora (C.) cayetanensis* in another single-tube multiplex real-time assay [3–5, 7, 8]. Additionally, PCR was used to detect soil-associated nematodes (*Ascaris (A.) lumbricoides*, *Ancylostoma* spp., *Necator (N.) americanus*, *Strongyloides (S.) stercoralis*) in a single-tube multiplex real-time assay [4, 9] and African *Schistosoma* spp. (*S. mansoni*, *S. intercalatum*, and *S. haematobium* without discrimination on species level) in a final simplex real-time assay [10, 11]. All in-house PCR procedures were performed after nucleic acid extraction using the QIAamp Stool DNA Mini Kit (Qiagen, Hilden, Germany) as described by the manufacturer and others [12]. Applied commercial PCRs comprised the RidaGene (R-Biopharm, Darmstadt, Germany) PCR kits “EAEC,”, “EHEC-EPEC”, and “‘ETEC-EIEC” targeting enteroaggregative *E. coli* (EAEC), enterohaemorrhagic *E. coli* (EHEC), enteropathogenic *E. coli* (EPEC), enterotoxic *E. coli* (ETEC), and *Shigella* spp./EIEC and were performed as described by the manufacturer.

If available, incidence data of the notifiable infectious diseases from the German population from the year 2015 were used for comparison. Such data are published in the yearly report by the Robert Koch Institute in the German National Reference Centre for Infectious Diseases. For the parameters assessed in the study, the respective reference data were available for enteroinvasive bacteria and EHEC, the enteropathogenic protozoa *G. duodenalis* and *Cryptosporidium* spp. as well as for the hepatitis B virus [13].

If refugees refused sample acquisition, their decision was accepted. Accordingly, not all patients submitted samples.

### Statistical assessment

The descriptive assessment of the data was performed using Microsoft Excel (Microsoft Corporation, Redmond, USA). Due to the small size of available samples and the absence of a study-specific sampling method, no further statistical calculations were performed for this descriptive assessment.

## Results

### Refugee characteristics

A total of 219 male UMRs between 13 and 18 years old were screened in our department between September 2014 and December 2015. The main countries of origin were Afghanistan (AFG) 92/219 (42.0%), Egypt (EGY) 48/219 (21.9%), Somalia (SOM) 24/219 (11.0%), Eritrea (ERI) 20/219 (9.1%), and Syria (SYR) 14/219 (6.4%), followed by Guinea (GIN) and Iran (IRN) with 4/219 (1.8%) each, Algeria (DZA) 3/219 (1.4%), Iraq (IRQ) 2/219 (0.9%), Benin (BEN), Gambia (GMB), Ghana (GHA), Libya (LBY), Morocco (MAR), Pakistan (PAK), and Palestine (PSE) with 1/219 (0.5%) each. For one UMR, the country of origin could not be determined. The detailed characteristics of the assessed UMRs with known country of origin are shown in Table 1.

**Table 1:** Age distribution per country or region of the assessed unaccompanied minor refugees (UMRs)

Item		AFG (*n* = 92)	EGY (*n* = 48)	ERI (*n* = 20)	SOM (*n* = 24)	SYR (*n* = 14)	Middle East (*n* = 8)	Other African countries (*n* = 12)
Gender (M, *n*)		92	48	20	24	14	8	12
Age (years)	Min.-Max.	13-18	14-17	15-17	14-17	15-17	15-17	15-17
	Mean	16.1	16	16.4	16.2	16.3	16.2	16.3
	SD	0.9	0.9	0.6	0.8	0.8	0.8	0.9
	Median	16.0	16.0	17.0	16.0	16.5	16.0	17.0

‘Middle East’ includes IRN, IRQ, PSE and PAK; ‘Other African countries’ includes GIN, LBY, BEN, DZA, GHA, MAR, and GMB; M. Male; SD. Standard deviation. For one UMR, the country of origin could not be determined. Only 218 out of 219 UMRs for whom information on their country of origin was available

### Screening results

#### Results of blood screenings

We obtained EDTA blood samples for cell counting from 218 UMRs. Serum samples for hepatitis serology were obtained from 190 refugees. For 10 of the UMRs, elevated eosinophil numbers of > 500 cells/μL were detected. The countries of origin of these UMRs included Afghanistan (6x), Eritrea (2x), Egypt (1x), and Guinea (1x). In one individual from Afghanistan and in another one from Eritrea, *Schistosoma* spp. were confirmed as the reason for eosinophilia, while the reason for eosinophilia could not be determined for the remaining 8 UMRs.

In serological analysis, HBs-antigen was detected in 3 individuals and anti-HBc-antibodies were found in 26 individuals. The countries of origin for these individuals are depicted in Table 2. While 3 out of 190 (1.6%) UMRs were HBs-antigen positive, the reported yearly incidence of hepatitis B in Germany in 2015 was 2.4 cases per 100,000 local residents [13].

**Table 2:** Country of origin of unaccompanied minor refugees (UMRs) that were positive for antibodies against hepatitis B core antigen (anti-HBc-antibodies) or hepatitis B surface antigen (HBs-antigen)

Country	Anti-HBc-antibodies	HBs-antigen
AFG	12/76 (15.8%)	0/76 (0%)
EGY	2/46 (4.3%)	1/46 (2.2%)
DZA	0/3 (0%)	0/3 (0%)
BEN	0/1 (0%)	0/1 (0%)
ERI	3/17 (17.6%)	0/17 (0%)
GHA	1/1 (100%)	0/1 (0%)
GIN	4/4 (100%)	1/4 (25%)
IRG	0/1 (0%)	0/1 (0%)
IRN	0/3 (0%)	0/3 (0%)
LBY	0/1 (0%)	0/1 (0%)
MAR	0/1 (0%)	0/1 (0%)
PAK	0/1 (0%)	0/1 (0%)
PSE	0/1 (0%)	0/1 (0%)
SOM	3/23 (13%)	1/23 (4.3%)
SYR	1/11 (9.1%)	0/11 (0%)
Total (%)	26/190 (13.7%)	3/190 (1.6%)

#### Results of stool screenings

Fixed stool samples for microscopy were provided by 158 UMRs, while non-fixed stool for PCR was provided by 157 UMRs. Interpretable results could be obtained by using PCR targeting invasive bacteria and enteropathogenic protozoa in 151 and 157 instances, respectively. We identified diarrhoea-associated *E. coli*/*Shigella* spp. and gut-associated helminths in 155 and 150 instances, respectively (Table 3).

**Table 3:** Detection of microorganisms in unaccompanied minor refugees (UMRs) by area of origin as well as by colonization, infestation and infection with multiple agents in UMRs by area of origin

	AFG	EGY	ERI	SOM	SYR	Other Middle Eastern countries	Other African countries	Total
UMRs from the assessed regions	n = 92	n = 48	n = 20	n = 24	n = 14	n = 8	n = 12	n = 218^a,b^
Diarrhoea-associated *E. coli* or *Shigella* spp. (PCR)	7/80 (8.8%) (EPEC = 4 EIEC/*Shigella* spp. = 1 EAEC = 2)	5/20 (25%) (EPEC = 5)	4/14 (28.6%) (EPEC = 2 EIEC/*Shigella* spp. = 1 EAEC = 1)	0/14 (0%)	2/10 (20%) (EPEC =1 EAEC = 1)	0/8 (0%)	1/9 (11.1%) (EAEC = 1)	19/155^b^ (12.3%)
Other enteroinvasive bacteria (PCR)	0/78 (0%)	0/20 (0%)	1/14 (7.1%) (*Campylobacter jejuni*)	0/14 (0%)	0/10 (0%)	0/7 (0%)	0/8 (0%)	1/151^b^ (0.7%)
*G. duodenalis* (microscopy and PCR)	12/82 (14.6%)	1/30 (3.3%)	3/16 (18.8%)	1/21 (4.8%)	0/9 (0%)	1/7 (14.3%)	2/10 (20%)	20/175^b^ (11.4%)
*Schistosoma* spp. (microscopy and PCR)	1/81 (1.2%)	0/29 (0%)	9/16 (56.3%)	1/21 (4.8%)	0/9 (0%)	0/7 (0%)	2/10 (20%)	13/173^b^ (7.5%)
Other helminths (microscopy and PCR)	3/81 (3.7%) (*Hymenolepis nana* = 2, *Strongyloides stercoralis* = 1)	0/29 (0%)	1/16 (6.3%) (*Strongyloides stercoralis* = 1)	1/21 (4.8%) (*Hymenolepis nana = 1)*	0/10 (0%)	0/7 (0%)	0/10 (0%)	5/174^b^ (2.9%)
*Blastocystis hominis* (microscopy)	38/74 (51.4%)	12/27 (44.4%)	12/16 (75%)	9/19 (47.4%)	0/6 (0%)	1/7 (14.3%)	5/11 (45.5%)	77/160^b^ (48.1%)
*Dientamoeba fragilis* (microscopy)	4/74 (5.4%)	2/27 (7.4%)	1/16 (6.3%)	0/19 (0%)	0/6 (0%)	1/7 (14.3%)	1/11 (9.1%)	9/160^b^ (5.6%)
*Endolimax nana* (microscopy)	3/74 (4.1%)	1/27 (3.7%)	2/16 (12.5%)	2/19 (10.5%)	0/6 (0%)	0/7 (0%)	0/11 (0%)	8/160 ^b^ (5%)
*Entamoeba coli* (microscopy)	4/74 (5.4%)	11/27 (40.7%)	2/16 (12.5%)	1/19 (5.3%)	0/6 (0%)	0/7 (0%)	1/11 (9.1%)	19/160^b^ (11.9%)
*Entamoeba hartmanni* (microscopy)	0/74 (0%)	2/27 (7.4%)	0/16 (0%)	2/19 (10.5%)	0/6 (0%)	0/7 (0%)	1/11 (9.1%)	5/160^b^ (3.1%)
*Entamoeba* spp. trophozoites / vegetative forms other than *E. histolytica* (microscopy)	5/74 (6.8%)	0/27 (0%)	0/16 (0%)	1/19 (5.3%)	0/6 (0%)	0/7 (0%)	1/11 (9.1%)	7/160^b^ (4.4%)
One microorganism detected	30/92 (32.6%)	10/48 (20.8%)	1/20 (5%)	10/24 (41.7%)	3/14 (21.4%)	3/8 (37.5%)	1/12 (8.3%)	58/218^b^ (26.6%)
Two microorganisms detected	15/92 (16.3%)	6/48 (12.5%)	4/20 (20%)	1/24 (4.2%)	0/14 (0%)	0/8 (0%)	4/12 (33.3%)	30/218^b^ (13.8%)
Three microorganisms detected	4/92 (4.3%)	0/48 (0%)	4/20 (20%)	2/24 (8.3%)	0/14 (0%)	0/8 (0%)	1/12 (8.3%)	11/218^b^ (5.0%)
Four microorganisms detected	4/92 (4.3%)	0/48 (0%)	3/20 (15%)	0/24 (0%)	0/14 (0%)	0/8 (0%)	0/12 (0%)	7/218^b^ (3.2%)
Five microorganisms detected	0/92 (0%)	0/48 (0%)	1/20 (5%)	0/24 (0%)	0/14 (0%)	0/8 (0%)	0/12 (0%)	1/218^b^ (0.5%)
Six microorganisms detected	0/92 (0%)	0/48 (0%)	0/20 (0%)	0/24 (0%)	0/14 (0%)	0/8 (0%)	1/12 (8.3%)	1/218^b^ (0.5%)

‘Middle East’ includes IRN, IRQ, PSE and PAK; ‘Other African countries’ includes GIN, LBY, BEN, DZA, GHA, MAR, GMB; ^a^For one UMR, the country of origin could not be identified. Accordingly, only 218 out of 219 UMRs are mentioned in this assessment. ^b^The denominators vary in a parameter-dependent way because not all assessments were performed for all UMRs

Not all results were available for all UMRs for technical reasons or because some UMRs denied approval to do some of the respective tests. Therefore, the denominators differ in a parameter-specific way

Among microbes of clinical and public health relevance, the combined PCR approach and microscopy identified 20 bacterial infections (comprising 12x EPEC, 5x EAEC, 2x EIEC/*Shigella* spp., and 1x *C. jejuni*), 20 *G. duodenalis* infections, and 18 helminth infections (comprising 13x *Schistosoma* spp., 3x *H. nana*, 2x *S. stercoralis*). Among the 20 cases with *G. duodenalis*, 5 (25%) were confirmed by microscopy and 18 by PCR (90%). Focusing on the two individuals with *G. duodenalis* identified by microscopy but not by PCR, in one instance, no sample was available for PCR. Among the 12 PCR-based detections of *Schistosoma* spp., 3 could be confirmed as *S. mansoni* by microscopy (Table 3). In one instance, the diagnosis occurred by microscopy only. The origins of the UMRs who tested positive for the above-mentioned pathogens are depicted in Table 3.

While 20 out of 175 (11.4%) of the assessed UMR samples were positive for *G. duodenalis*, the reported yearly incidence in the local residents in 2015 was only 4.5 cases per 100,000 inhabitants [13]. While 1 out of 151 (0.7%) of the assessed stool samples were positive for *C. jejuni*, the reported yearly incidence of *Campylobacter*-induced enteritis in 2015 was 87 cases per 100,000 inhabitants in Germany [13]. Finally, while 2 out of 155 assessed samples were positive for EIEC/*Shigella* spp., the reported yearly incidence of shigellosis in Germany in 2015 was 0.7 cases per 100,000 inhabitants [13].

Following the enteric pathogens, colonization by protozoa also indicates poor food and water hygiene conditions. We identified high percentages of the following (Table 3) using microscopy: *Blastocystis (B.) hominis*, *Endolimax (E.) nana*, *Entamoeba (E.) coli*, *E. hartmanni*, additional *Entamoeba* spp. trophozoites/vegetative forms other than *E. histolytica* as well as *Dientamoeba (D.) fragilis* (Table 3).

#### Multiple colonizations, infestations and infections

Multiple colonizations, infestations or infections with multiple pathogenic microorganisms, as well as microorganisms indicating a lack of proper hygiene, were frequently observed (Table 4) with varying frequencies depending on the country of origin. Up to six different microorganisms were identified per individual (Table 3).

**Table 4:** Matrix of the combined detections of microorganisms of relevance because of their clinical or public health significance in the assessed unaccompanied minor refugees (UMRs)

	*G. duodenalis* *n* = 20	*S. stercoralis* *n* = 2	*S. mansoni* *n* = 13	*H. nana* *n* = 3	*C. jejuni* *n* = 1	anti-HBc-antibodies *n* = 26	HBs-antigen *n* = 3	EPEC *n* = 12	EIEC *n* = 2	EAEC *n* = 5
*G. duodenalis* *n* = 20		1/2 (50%)	5/13 (38.5%)	0/3 (0%)	0/1 (0%)	4/26 (15.4%)	0/3 (0%)	1/12 (8.3%)	0/2 (0%)	1/5 (20%)
*S. stercoralis* *n* = 2	1/20 (5%)		1/13 (7.7%)	0/3 (0%)	0/1 (0%)	0/26 (0%)	0/3 (0%)	0/12 (0%)	0/2 (0%)	0/5 (0%)
*S. mansoni n* = 13	5/20 (25%)	1/2 (50%)		0/3 (0%)	1/1 (100%)	5/26 (19.2%)	1/3 (33.3%)	1/12 (8.3%)	0/2 (0%)	1/ 5 (20%)
*H. nana* *n* = 3	0/20 (0%)	0/2 (0%)	0/13 (0%)		0/1 (0%)	0/26 (0%)	0/3 (0%)	0/12 (0%)	0/2 (0%)	0/5 (0%)
*C. jejuni* *n* = 1	0/20 (0%)	0/2 (0%)	1/13 (7.7%)	0/3 (0%)		0/26 (0%)	0/3 (0%)	0/12 (0%)	0/2 (0%)	0/5 (0%)
anti-HBc- *n* = 26	2/20 (10%)	0/2 (0%)	5/13 (38.5%)	0/3 (0%)	0/1 (0%)		3/3 (100%)	0/12 (0%)	1/2 (50%)	1/5 (20%)
HBs-antigen *n* = 3	0/3 (0%)	0/2 (0%)	1/13 (7.7%)	0 /3 (0%)	0/1 (0%)	3/26 (11.5%)		0/12 (0%)	0/2 (0%)	0/5 (0%)
EPEC *n* = 12	1/20 (5%)	0/2 (0%)	1/13 (7.7%)	0/3 (0%)	0/1 (0%)	0/26 (0%)	0/3 (0%)		0/2 (0%)	1/5 (20%)
EIEC *n* = 2	0/20 (0%)	0/2 (0%)	0/13 (0%)	0/3 (0%)	0/1 (0%)	1/26 (3.8%)	0/3 (0%)	0/12 (0%)		0/5 (0%)
EAEC *n* = 5	1/20 (5%)	0/2 (0%)	1/13 (7.7%)	0/3 (0%)	0/1 (0%)	1/26 (3.8%)	0/3 (0%)	1/5 (20%)	0/2 (0%)	

All assessments of UMRs were negative for *C. parvum*, *E. histolytica*, *C. cayetanensis*, *Shigella* spp./EIEC, *C. jejuni*, *Yersinia* spp., soil-associated nematodes (*A. lumbricoides*, *Ancylostoma* spp., *N. americanus*, *S. stercoralis*), EHEC, and ETEC

When comparing co-colonization with different pathogenic microorganisms (Table 4), co-incidence of *G. duodenalis* and *Schistosoma* spp. detections was most frequently observed.

## Discussion

Infectious diseases are the dominant disease burdens in UMRs coming to Germany, as shown by a previous cross-sectional study that showed a prevalence of infection of 58.8%. UMRs from Sub-Saharan Africa were most frequently infected at a percentage of 86.7% [14].

The most frequent infectious diseases that were associated with outbreaks in centralized homes for asylum seekers in Germany are chicken pox (30%), measles (20%), scabies (19%) as well as rota-virus-gastroenteritis (8%) [15]. These infections demonstrate the importance of appropriate hygiene protocols. Mass vaccination against measles was identified to be a cost-efficient approach to prevent outbreaks [16], but it requires a sufficient capacity of qualified personnel to cope with the sudden rises in patient numbers, especially in young refugees. Immediate vaccination without prior serological testing for pre-existing antibodies was performed in line with local standards [17] after the exclusion of medical contradictions and appropriate medical consulting for the UMRs.

The present analysis of the UMR screening results suggests considerable infestation, infection and colonization with both pathogenic microorganisms and microorganisms that are indicative of living under poor hygiene conditions, such as various protozoa. As shown in the country distributions and as previously suggested [14], the infectious burden seemed highest in UMRs from Sub-Saharan Africa with an overall dominance of commensal microorganisms. However, the low number of UMRs from Sub-Saharan Africa in this assessment did not allow for a statistically sound confirmation of this observation. Pathogenic *E. coli*, *G. duodenalis* and *Schistosoma* spp. comprised the most frequently detected pathogens.

Similar reports about refugees coming to Germany have been provided by other authors as well. The results by Heudorf et al. [18] showed similar detection rates for *G. duodenalis* and *H. nana* in UMRs but a considerably lower infestation rate with *Blastocystis* spp. These authors also detected the presence of *Trichuris trichiura* and *E. histolytica*, which were completely absent in our assessment. When focusing on the Syrian UMRs only, who were quantitatively underrepresented in our assessment, Mockenhaupt et al. [19] reported a lower detection rate of 7% infection with *G. duodenalis*, positive schistosomiasis serology in 1.4% of UMRs and the absence of hepatitis B [19]. In our study, colonization with diarrhoea-associated *E. coli* comprised the only diagnostic result that had potential aetiological relevance in the 14 assessed Syrian UMRs. In clinically ill adult refugees from Syria, cutaneous leishmaniasis, active tuberculosis, as well as hepatitis B and C were quantitatively dominant [19]. High seroprevalence of hepatitis is generally expected in refugees from high endemicity settings [20], which was confirmed by our analysis. The low rate of HBs-antigen detection among the anti-HBc-positive UMRs in our study suggests that transmission routes other than vertical transmission are likely.

Surveillance regarding latent tuberculosis and especially latent multi-drug resistant tuberculosis in migrants coming to Germany deserves further consideration [21]. Available point prevalence data seems to reflect a similar prevalence of tuberculosis in refugees as in their countries of origin [22], which is usually higher than in Germany. It is also expected that there is also an increased risk of exposure to multi-drug resistant tuberculosis for German health care workers who care for migrated patients [23]. In the small group of UMRs assessed here, no cases of active tuberculosis were identified by chest x-ray, which is a standard screening tool for active pulmonary tuberculosis for migrants coming to Germany. Although some authors describe that the sensitivity of gamma-interferon release assays is higher than that of chest x-ray for the diagnosis of active pulmonary tuberculosis in sputum-negative patients [24], a recent literature review indicated that chest x-rays have acceptable to good sensitivity for the diagnosis of pulmonary tuberculosis [25]. Further, the positive predictive value of gamma-interferon release assays for the diagnosis of active tuberculosis is low for patients from high-endemicity settings because positive results can also be expected in cases of latent or cured tuberculosis [24].

The results presented in this study impressively demonstrate that the diagnostic in-house real-time PCR systems, which were established and evaluated for soldiers returning from tropical deployments, are also suitable for the screening of refugees. For soldiers who must operate on their own under tropical conditions with restricted hygiene conditions, *G. duodenalis* [3] and diarrhoea-associated *E. coli* [5] are among the most frequently detected pathogens. As shown for European soldiers in West African Mali, frequently detected EPEC may play an etiologically relevant role in adult patients with diarrhoea [5], meaning that the affected soldiers are not adapted to this pathogen due to the good hygiene in their home-countries.

The fact that PCR is much more sensitive than microscopy for the detection of protozoa in stool samples is a well-known phenomenon [11]. Admittedly, not all laboratories have a broad range of in-house PCRs, and commercial syndromic stool assays are often expensive and are, therefore, usually out of range for public health services. Appropriately evaluated in-house PCR approaches are a recommended option. If in-house approaches are used, such as in the study presented here, reagent costs are no more than 10–15 Euro per sample, including the nucleic acid extraction procedure.

The fact that the screened UMRs did not report symptoms of diarrhoea confirms that the presence of enteropathogenic microorganisms in the stool of individuals arriving from high-prevalence settings is not necessarily associated with clinical disease. This phenomenon is known from previous assessments. Even multiple detections of pathogens in a single individual are not necessarily associated with clinical disease [4, 5]. Nevertheless, except for *Schistosoma* spp., in this study, all the PCR-based detected microorganisms with pathogenic potential are transmitted via the faecal-oral route and are thus problematic for hygiene management, particularly in overcrowded accommodations. Finally, the detection of multiple colonization events further suggests that any positive screening result should lead to looking more intensively for additional pathogens.

## Conclusion

In summary, the results of this study can be summarized as follows. 1. In-house PCR screening systems, which are useful for military returning from tropical deployments, can also be applied to civilian migrants from their respective states of origin. 2. Apparently healthy UMRs may be enterically colonized with a broad variety of pathogenic and non-pathogenic microorganisms, which can pose a hygiene problem in reception centres. As demonstrated for infectious agents that are notifiable in Germany, such as *G. duodenalis*, higher detection rates should be expected in UMRs. Most of the detected pathogens can be transmitted via the faecal-oral route. 3. Colonization with multiple pathogens occurs. Therefore, incidental detection of a single pathogen might suggest the presence of other ones as well.

## Abbreviations

*A. lumbricoides*: *Ascaris lumbricoides*
AFG: Afghanistan
BEN: Benin
BNITM: Bernhard Nocht Institute for Tropical Medicine
BwKH: German Armed Forces Hospital Hamburg
*C. cayetanensis*: *Cyclospora cayetanensis*
*C. jejuni*: *Campylobacter jejuni*
*C. parvum*: *Cryptosporidium parvum*
*D. fragilis*: *Dientamoeba fragilis*
DTM-BNITM: Department of Tropical Medicine at the Bernhard Nocht Institute
DZA: Algeria
*E. coli*: *Escherichia coli*
*E. hartmannii*: *Entamoeba hartmannii*
*E. histolytica*: *Entamoeba histolytica*
*E. nana*: *Endolimax nana*
EAEC: Enteroaggregative *Escherichia coli*
EGY: Egypt
EHEC: Enterohemorrhagic *Escherichia coli*
EIEC: Enteroinvasive *Escherichia coli*
EPEC: Enteropathogenic *Escherichia coli*
ERI: Eritrea
ETEC: Enterotoxic *Escherichia coli*
*G. duodenalis*: *Giardia duodenalis*
GHA: Ghana
GIN: Guinea
GMB: the Gambia
*H. nana*: *Hymenolepis nana*
HBc: *H*epatitis B core antigen
HBs: Hepatitis B surface antigen
IRN: Iran
IRQ: Iraq
LBY: Libya
MAR: Morocco
*N. americanus*: *Necator americanus*
PAK: Pakistan
PCR: Polymerase chain reaction
PSE: Palestine
*S. haematobium*: *Schistosoma haematobium*
*S. intercalatum*: *Schistosoma intercalatum*
*S. mansoni*: *Schistosoma mansoni*
*S. stercoralis*: *Strongyloides stercoralis*
SD: Standard deviation
SOM: Somalia
spp.: Species (plural)
SYR: Syria
UMR: Unaccompanied minor refugee
μl: Micro-liter
